# Characterization of Argentine honeys on the basis of their mineral content and some typical quality parameters

**DOI:** 10.1186/1752-153X-8-44

**Published:** 2014-07-02

**Authors:** Marcelo Enrique Conti, Maria Grazia Finoia, Luca Fontana, Giustino Mele, Francesco Botrè, Ivo Iavicoli

**Affiliations:** 1 Department of Management, University of Rome, Sapienza, Via del Castro Laurenziano 9, Rome 00161, Italy; 2 Italian National Institute for Environmental Protection and Research, Viale V. Brancati 60, Rome 00166, Italy; 3 Institute of Occupational Medicine, Università Cattolica del Sacro Cuore, Largo Francesco Vito 1, Rome 00168, Italy; 4 Department of Experimental Medicine, University of Rome, Sapienza, Viale Regina Elena 324, Rome 00161, Italy

**Keywords:** Honey, Elements’ content, Quality parameters, Multivariate statistical tools, Cluster analysis, Pattern recognition methods, Environmental biomonitoring

## Abstract

**Background:**

The levels of 19 elements (As, Be, Ca, Cd, Co, Cr, Cu, Fe, K, Mg, Mn, Na, Ni, Pb, Se, Tl, U, V, Zn) from sixteen different Argentine production sites of unifloral [eucalyptus (*Eucaliptus rostrata*), chilca (*Baccharis salicifolia*), Algarrobo (*Prosopis sp.*), mistol (*Ziziphus mistol*) and citric] and multifloral honeys were measured with the aim to test the quality of the selected samples. Typical quality parameters of honeys were also determined (pH, sugar content, moisture). Mineral elements were determined by using inductively coupled plasma mass spectrometer (ICP-MS DRC). We also evaluated the suitability of honey as a possible biomonitor of environmental pollution. Thus, the sites were classified through cluster analysis (CA) and then pattern recognition methods such as Principal Component Analysis (PCA) and discriminant analysis (DA) were applied.

**Results:**

Mean values for quality parameters were: pH, 4.12 and 3.81; sugar 82.1 and 82.0 °brix; moisture, 16.90 and 17.00% for unifloral and multifloral honeys respectively. The water content showed good maturity. Likewise, the other parameters confirmed the good quality of the honeys analysed. Potassium was quantitatively the most abundant metal, accounting for 92,5% of the total metal contents with an average concentration of 832.0 and 816.2 μg g^-1^ for unifloral and multifloral honeys respectively. Sodium was the second most abundant major metal in honeys with a mean value of 32.16 and 33.19 μg g^-1^ for unifloral and multifloral honeys respectively. Mg, Ca, Fe, Mn, Zn and Cu were present at low-intermediate concentrations. For the other 11 trace elements determined in this study (As, Be, Cd, Co, Cr, Ni, Pb, Se, Tl, U and V), the mean concentrations were very low or below of the LODs. The sites were classified through CA by using elements’ and physicochemical parameters data, then DA on the PCA factors was applied. Dendrograms identified three main groups. PCA explained 52.03% of the total variability with the first two factors.

**Conclusions:**

In general, there are no evidences of pollution for the analysed honeys. The analytical results obtained for the Argentine honeys indicate the products’ high quality. In fact, most of the toxic elements were below LODs. The chemometric analysis combining CA, DA and PCA showed their aptness as useful tools for honey’s classification. Eventually, this study confirms that the use of honey as biomonitor of environmental contamination is not reliable for sites with low levels of contamination.

## Background

Honey is defined as “the natural sweet substance produced by *Apis mellifera* bees from the nectar of plants or from secretions of living parts of plants or excretions of plant-sucking insects on the living parts of plants, which the bees collect, transform by combining with specific substances of their own, deposit, dehydrate, store and leave in honeycombs to ripen and mature” [1]. Argentina is one of the major producers of bee honey and is the leading global exporter of high quality honey. About 50% come from the Province of Buenos Aires [2,3]. For instance, the United States imports 19% of their foreign honey from Argentina, spending about 54 million dollars in 2010. Also European countries such as Italy and Germany are strongly influenced by competition from Argentine and Chinese varieties whose prices are lower by roughly 50% [4].

Honey has high nutritional value (330 kcal/100 g) and fast absorption of its carbohydrates on consumption. It is a high carbohydrate food and shows anti-bacterial and anti-inflammatory properties in the treatment of skin wounds and several gastrointestinal diseases [4-10]. Honey activates the immune system and its ingestion may be beneficial with respect to cancer and metastasis prevention [5,10]. Hydrogen peroxide produced enzymatically is responsible for honey’s antibacterial activity [7]. Honey is a potent inhibitor of the bacterium *Helicobacter pylori* that causes peptic ulcers and gastritis [10].

The European Union [1] defines general and specific compositional characteristics of honey such as sugar content, humidity, acidity, electrical conductivity, diastase activity and hydroxymethylfurfural (HMF) content. Moreover, labels on honey packaging should report information on the product’s regional or topographical origin, floral or vegetable origin. If honey originates from different countries the label should specify “blend of EU (or no-EU) honeys” or “blend of EU and no-EU honeys”.

The composition and properties of a particular honey sample depend highly on the type of flowers visited by the bees, as well as on the climatic conditions in which the plants grow [11,12]. Melissopalynology (pollen analysis) is the traditional method used to determine the botanical origin of honeys [13,14], but this technique has some limitations [15]. In fact, melissopalynology requires relevant knowledge of pollen morphology and specialised professional personnel to achieve reliable results [13]. However, nowadays in spite of these problems melissopalynology remains the reference method.

Bees forage an area of about 7 km^2^ and came in contact constantly with the surrounding environment. The chemical composition and properties of honey depend on the type of flowers visited by the bees, as well as on the climatic conditions in which the plants grow [4,9]. This also implies that honeybees and their products (i.e. pollen, wax, etc.) can be employed as potential biomonitors of environmental contamination [9,16]. Finally, the specific chemical and physical properties can be used for the determination of the botanical origin of honey [17,18].

The role of elements in honey is of high relevance [19-21] in terms of both its quality and safety. Scarce information is available on the elements’ composition of Argentine honeys, this is also connected with the relevance of Argentina as one of the main honey exporter in the world. In fact, only 5% of the total honey production in Argentina is destined to domestic consumption.

The aim of the work was to measure the levels of 19 elements (As, Be, Ca, Cd, Co, Cr, Cu, Fe, K, Mg, Mn, Na, Ni, Pb, Se, Tl, U, V, Zn) and some typical quality parameters (pH, sugar content, moisture) from sixteen different Argentine production sites of unifloral [i.e. eucalyptus, chilca (*Baccharis* spp.), Algarrobo (*Prosopis* sp.), mistol (*Ziziphus mistol*) and citric] and multifloral honey samples. Duplicate samples were taken from each production area. Due to its economic relevance for export purposes and for production levels, samples were mainly collected in the Province of Buenos Aires.

We have evaluated, by means of multivariate statistical methods whether the physicochemical parameters and the elements’ content can classify or discriminate the sampling sites in order to confirm the suitability of honey as a possible biomonitor of environmental pollution. Thus, the sites were classified through cluster analysis by using elements’ data, then pattern recognition methods such as Principal Component Analysis (PCA) and discriminant analysis (DA) were applied (see Statistical methods section for details).

## Results and discussion

Table 1 reports the mean ± SD and the range of the physicochemical parameters for the analysed honeys. Reported data for the physicochemical parameters were homogeneous for the analyzed honeys, showing very low SD levels (Table 1).

**Table 1:** Descriptive statistics for physicochemical parameters in Argentine honey samples

	**N**	**Mean** **±** **SD**	**MIN**	**MAX**
**SUGAR [°brix]**				
Unifloral	5	82.1 ± 0.7	81.5	83.0
Multifloral	11	82.0 ± 1.2	80.5	84.0
**pH**				
Unifloral	5	4.12 ± 0.21	3.87	4.46
Multifloral	11	3.81 ± 0.27	3.55	4.43
**MOISTURE [%]**				
Unifloral	5	16.9 ± 0.6	16.0	17.5
Multifloral	11	17.0 ± 1.3	15.0	19.0

The pH ranged between 3.87 and 4.46 with a mean value of 4.12 for unifloral honeys, while the range was between 3.55 and 4.43 with a mean of 3.81 for multifloral honeys.

Most bacteria and moulds grow in a neutral and mildly alkaline environment respectively, while yeasts require an acidic environment (pH = 4.0 – 4.5) and do not grow in alkaline media [22]. In fact, pH is a useful index of possible microbial contamination [23] and has great relevance during the extraction and storage of honey because it is connected with the texture and the product’s shelf life [24].

The mean pH value (3.81) of multifloral Argentine honeys was similar than those reported by Baroni et al. [25] for Córdoba (Argentina) honeys and by Conti et al. [4] for Italian honeys (Marche Region); while the obtained mean pH value for unifloral honeys (i.e. 4.12) was comparable to those of Lazio Region (Italy) honeys [24] and to Andalusian (Spain) unifloral honeys [26].

Water content is connected with the climatic conditions and the degree of maturity; anomalous values may be an index of adulterations. It generally depends on the botanical origin of the sample, the processing techniques and the storage conditions [4,24,27]. Mean humidity was 17.0% with a range of between 15.0 – 19.0% for multifloral honeys and a mean of 16.9% with a range of between 16.0 – 17.5% for unifloral honeys. None of the samples exceeded the limit permitted of 20% by the Codex Alimentarius [28] and the Council Directive [1]. However one sample (code M8, Entre Rios Province) showed a value higher than the limit of 18% established by the Argentine legislation [29]. Our results generally confirm that the fermentation rate is very low in the analyzed samples. Moisture values observed for our samples were slightly lower than those obtained for Córdoba (Argentina) honeys [25] and quite similar to honeys from Buenos Aires Region [30], and to those of Andalusia (Spain) [26], Lazio Region (Italy) [24], Serbian Acacia honeys [27] and to *Entre-Douro* e *Minho* Region of Portugal [31].

The average total sugar content was 82.1% and the range was 81.5 – 83.0% for unifloral honeys, and a mean of 82.0% with a range of between 80.5 – 84.0% for multifloral honeys. Our results match those obtained for Italian and Spanish honeys [4,23,24,26].

Tables 2 and 3 show the mean, standard deviation and the concentration ranges of the elements for unifloral and multifloral honey samples respectively.

**Table 2:** **Descriptive statistics of elements’ content (μg g**^**-1 **^**wet weight) in Argentine unifloral honey samples**

	**Eucalipto (EntreRios)**	**Chilca (Còrdoba)**	**Algarrobo (S. del Estero)**	**Mistol (S. del Estero)**	**Citric (Tucumàn)**	**Mean± SD**	**Min**	**Max**
	**M1**	**M2**	**M3**	**M4**	**M5**			
**As**	<0.01	<0.01	<0.01	<0.01	<0.01	-	<0.01	<0.01
**Be**	<0.01	<0.01	<0.01	<0.01	<0.01	-	<0.01	<0.01
**Ca**	6.18	2.50	4.13	13.74	8.04	6.92±4.35	2.50	13.74
**Cd**	<0.01	<0.01	<0.01	0.01	<0.01	0.01±0.00	<0.01	0.01
**Co**	0.01	<0.01	<0.01	<0.01	<0.01	0.01±0.00	<0.01	0.01
**Cr**	<0.01	<0.01	<0.01	<0.01	<0.01	-	<0.01	<0.01
**Cu**	0.27	0.12	0.20	0.18	0.15	0.18±0.06	0.12	0.27
**Fe**	3.38	2.19	4.05	3.73	4.50	3.57±0.87	2.19	4.50
**K**	488.4	251.1	318.4	2022.6	1079.8	832.0±741.3	251.1	2022.6
**Mg**	14.37	4.59	8.12	37.98	21.26	17.26±13.21	4.59	37.98
**Mn**	8.84	0.77	1.14	1.60	0.70	2.61±3.50	0.70	8.84
**Na**	62.00	7.21	39.27	34.65	17.67	32.16±21.09	7.21	62.00
**Ni**	0.05	0.01	0.06	0.02	0.03	0.04±0.02	0.01	0.06
**Pb**	0.01	0.02	0.02	0.01	0.02	0.02±0.01	0.01	0.02
**Se**	0.01	<0.01	<0.01	0.01	<0.01	0.01±0.00	0.01	0.01
**Tl**	<0.01	<0.01	<0.01	<0.01	<0.01	-	<0.01	<0.01
**U**	<0.01	<0.01	<0.01	<0.01	<0.01	-	<0.01	<0.01
**V**	<0.01	<0.01	<0.01	<0.01	<0.01	-	<0.01	<0.01
**Zn**	0.55	1.05	1.42	0.84	0.51	0.87±0.38	0.51	1.42

**Table 3:** **Descriptive statistics of elements’ content (μg g**^**-1 **^**wet weight) in Argentine multifloral honey samples**

	**Còrdoba**	**Prov. Buenos Aires**	**Entre Rios**	**Entre Rios**	**Prov. Buenos Aires**	**Prov. Buenos Aires**	**Prov. Buenos Aires**	**Prov. Buenos Aires**	**Prov. Buenos Aires**	**Prov. Buenos Aires**	**Prov. Buenos Aires**	**Mean ± SD**	**Min**	**Max**
	**M6**	**M7**	**M8**	**M9**	**M10**	**M11**	**M12**	**M13**	**M14**	**M15**	**M16**			
**As**	<0.01	<0.01	<0.01	0.01	<0.01	<0.01	<0.01	<0.01	<0.01	<0.01	<0.01	0.01 ± 0.00	<0.01	0.01
**Be**	<0.01	<0.01	0.10	<0.01	<0.01	<0.01	<0.01	<0.01	<0.01	<0.01	<0.01	0.10 ± 0.00	<0.01	0.10
**Ca**	7.57	6.03	17.26	18.97	15.33	14.68	13.61	18.61	2.93	1.97	2.50	10.86 ± 6.75	1.97	18.97
**Cd**	<0.01	<0.01	<0.01	0.03	0.01	0.01	<0.01	<0.01	<0.01	<0.01	<0.01	0.02 ± 0.01	<0.01	0.03
**Co**	<0.01	<0.01	0.01	0.01	<0.01	0.01	<0.01	<0.01	<0.01	0.01	<0.01	0.01 ± 0.00	<0.01	0.01
**Cr**	<0.01	0.01	0.01	<0.01	<0.01	<0.01	0.05	0.04	<0.01	<0.01	<0.01	0.03 ± 0.02	<0.01	0.05
**Cu**	0.13	0.09	0.38	1.19	0.17	0.20	0.12	0.32	0.20	0.17	0.21	0.29 ± 0.31	0.09	1.19
**Fe**	4.40	2.07	3.15	2.24	2.32	4.18	2.26	3.99	2.40	3.22	2.68	2.99 ± 0.86	2.07	4.40
**K**	1225.8	324.9	1408.2	2813.3	755.8	476.2	412.3	1107.5	176.6	134.1	143.9	816.2 ± 802.6	134.1	2813.3
**Mg**	18.12	12.39	75.38	49.77	19.68	23.57	16.46	23.87	3.43	3.01	3.31	22.64 ± 21.95	3.01	75.38
**Mn**	0.57	0.55	2.39	3.13	1.18	0.57	0.14	0.40	0.60	1.40	0.59	1.05 ± 0.93	0.14	3.13
**Na**	20.05	4.88	36.30	28.62	105.95	25.19	25.79	76.44	13.26	15.45	13.16	33.19 ± 30.66	4.88	105.95
**Ni**	0.02	0.02	0.07	0.05	0.02	0.05	0.03	0.04	0.03	0.05	0.02	0.04 ± 0.02	0.02	0.07
**Pb**	0.01	0.04	0.01	0.01	0.04	0.04	0.01	0.04	0.01	0.03	0.01	0.02 ± 0.02	0.01	0.04
**Se**	<0.01	<0.01	<0.01	<0.01	<0.01	<0.01	<0.01	<0.01	<0.01	<0.01	<0.01	-	<0.01	<0.01
**Tl**	<0.01	<0.01	<0.01	<0.01	<0.01	<0.01	<0.01	<0.01	<0.01	<0.01	<0.01	-	<0.01	<0.01
**U**	<0.01	<0.01	<0.01	<0.01	<0.01	<0.01	<0.01	<0.01	<0.01	<0.01	<0.01	-	<0.01	<0.01
**V**	<0.01	<0.01	<0.01	<0.01	<0.01	<0.01	<0.01	<0.01	<0.01	<0.01	<0.01	-	<0.01	<0.01
**Zn**	2.75	1.22	0.80	0.70	0.81	2.04	1.50	0.74	0.72	0.92	0.70	1.17 ± 0.67	0.70	2.75

Potassium was quantitatively the most abundant metal, accounting for 92,5% of the total metal contents with an average concentration of 832.0 and 816.2 μg g^-1^(wet weight) for unifloral and multifloral Argentine honeys respectively. For data comparison, the results were appropriately transformed (i.e. wet or dry basis) when necessary.

As above pointed out, little information is available on mineral content in Argentine honeys. Our mean potassium levels were considerably higher than those of Córdoba (Argentina) honeys [25] and to Lazio (Italy) honeys [24] and higher to Marche region honeys (Italy) [4]. This result is consistent with other reported data [9,19,32].

Sodium, as expected, was the second most abundant major metal in honeys. We determined a mean sodium content of 32.16 and 33.19 μg g^-1^ (w.w.) for unifloral and multifloral Argentine honeys respectively. Mean sodium values were lower than Córdoba (Argentina) honeys [25] and Lazio (Italy) honeys [24]; while we obtained slightly higher mean values than those reported for Marche region (Italy) honeys [4].

Mean magnesium levels were 17.26 and 22.64 μg g^-1^w.w. in unifloral and multifloral Argentine honeys respectively, and were quite higher than those determined in Córdoba (Argentina) honeys [25]. Calcium mean levels, i.e. 6.92 and 10.86 μg g^-1^ w.w, for unifloral and multifloral Argentine honeys respectively, were lower than Córdoba (Argentina) honeys [25], than to Spanish [33], and to Lazio and Marche Regions (Italy) honeys [4,24]. The mean iron levels in our samples, 3.57 and 2.99 μg g^-1^ w.w. for unifloral and multifloral Argentine honeys respectively, were lower than those of Córdoba (Argentina) [25], to San Luis – La Pampa honeys (middle Argentina) [34] and to Lazio and Marche Regions (Italy) honeys [4,24].

The mean manganese levels (2.61 and 1.05 μg g^-1^w.w.) were higher to those found for Córdoba (Argentina) honeys [25], and to Marche region (Italy) honeys [4], and lower than Lazio region (Italy) honeys [24]. The mean zinc levels (0.87 and 1.17 μg g^-1^ w.w.) were lower than those found for Córdoba (Argentina) honeys [25] and Lazio honeys [24] and comparable to those found for San Luis – La Pampa honeys (middle Argentina) [34].The mean copper levels for our samples (0.18 and 0.29 μg g^-1^) are lower than those of Lazio honeys [24] while Cu was not detected in the Còrdoba (Argentina) honeys [25].

Potassium showed positive correlation with Ca [r = 0.70 t(14) = 3.68 p = 0.002], Mg [r = 0.76 t(14) = 4.44 p < 0.001] and Cu [r = 0.73 t(14) = 4.04 p = 0.001]. Calcium correlated positively with Mg [r = 0.77 t(14) = 4.53 p < 0.001] and Cu [r = 0.50 t(14) = 2.17 p = 0.047]. Sodium correlated positively with Ca [r = 0.524 t(14) = 2.30 p = 0.037].

The presence of these major essential elements in honey such as Ca, K, Na and Mg is of certain nutritional relevance and dietary value, mainly connected with children’s health [9,19]. As previously reported [24] the intake of many major and minor metals (i.e. Fe, Mn, Cu and Zn) from honey is very low due to its low level of consumption. Generally, honey does not contribute for a significant proportion of minerals recommended dietary allowances (RDAs), usually from a few per cent, or even lower [24,35].

For the other 11 trace elements determined in this study (As, Be, Cd, Co, Cr, Ni, Pb, Se, Tl, U and V), the mean concentrations were very low or below of the LODs (Tables 2 and 3). In general, the presence of these elements can indicate contamination during honey processing, shipping or storage connected with the use of steel or galvanized containers [19]. However, in an another study chromium levels in some honey samples collected in the Buenos Aires province (Argentina) were in the range of 0.9 – 6 μg g^-1^[36] while a mean concentration of 0.47 μg g^-1^ is reported for San Luis – La Pampa honeys (middle Argentina) [34].

Lead levels were lower than others obtained for honeys collected in the Buenos Aires province (Argentina) [36], and comparable to those reported for Turkish honeys [37], while our nickel levels were higher than those of Turkish honeys [37]. Generally, with rare exceptions, our data for some trace elements (i.e. As, Cd, Co, Cr, Pb) are comparable or at a lower levels than those reported for other countries [38-42]. Very scarce information is present in literature about some elements in Argentine honeys such us Ni, Se, Tl, U and V. Our selenium levels were lower than those measured in Turkish honeys [37]. While we not detected uranium and vanadium in our samples (LOD < 0.01 μg g^-1^, Tables 2 and 3) Almeida-Silva et al. [43] reported levels of 0.34 and 0.28 μg g^-1^ of U and 13.5 and 5.6 μg g^-1^ of vanadium in Portuguese honeys.

We have no found data for beryllium in honeys in the literature. However, we detected Be only in the Entre Ríos (M8) multifloral sample (0.10 μg g^-1^) while the others were below LODs. At present, there are no studies that suggested a health risk for the presence of beryllium in food and drinking water, even if it can be an index of industrial contamination [44].

Overall, from our results we can infer that there are no evidences of pollution for the analysed honeys and these results confirm their good quality.

**Figure 1:**
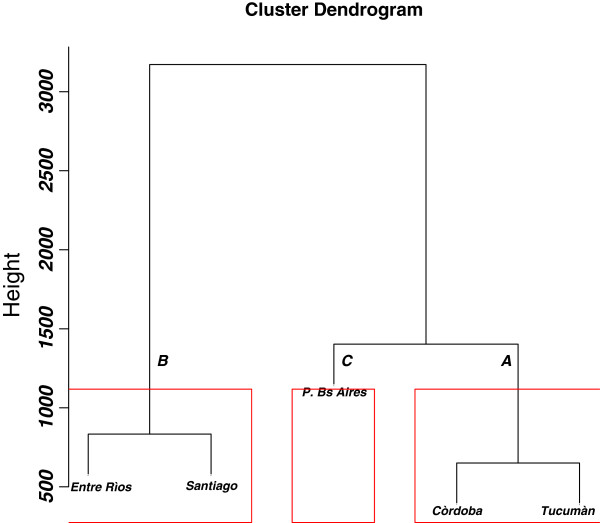
Hierarchical cluster analysis.

**Figure 2:**
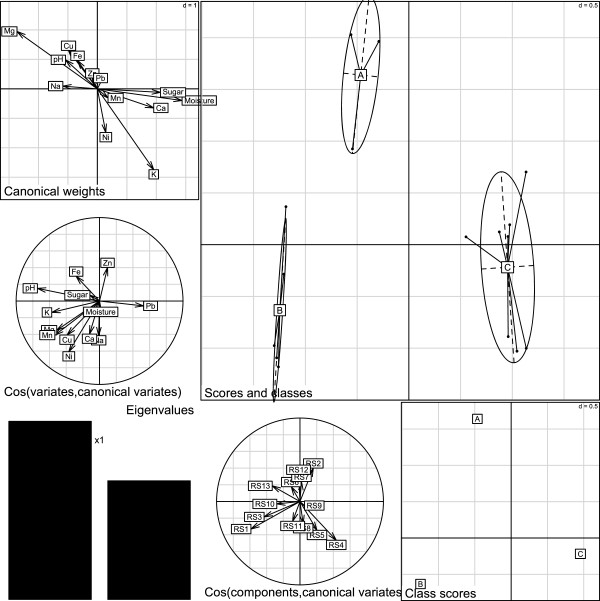
**DA on PCA factors for honeys’ elements data and physicochemical parameters applied for the Hierarchical Cluster Analysis results.** Composed plot; (top left): the plot of the canonical weights; (middle left) the plot of canonical correlations between variates and the first two canonical discriminant functions; (bottom left): the eigenvalues bar chart; (bottom centre): the plot of PCA factors into LDA plane; (bottom right): the gravity centers of classes; [main graph]: the projection of the canonical scores with ellipses and gravity center of classes.

We applied the hierarchical clustering by using data set (Tables 1, 2 and 3, see also Experimental section below) in order to classify the production sites. The result of the cluster analysis is reported in Figure 1. The x axis depicts the sampling sites while the y axis indicates the calculated distances among sampling sites. From Figure 1 we observe that there are three main groups. The honeys produced in the provinces of Córdoba and Tucumán are grouped in the A cluster. The honeys produced in the provinces of Entre Ríos and Santiago (i.e. Santiago del Estero) are grouped in the B cluster, and those produced in the Buenos Aires province are linked with the A cluster at the last step of the iterative aggregation process.Thus, in order to discriminate the elements and parameters classified by cluster analysis, we applied discriminant analysis (DA) on principal component analysis (PCA) factors (see experimental section). PCA explained 52.03% of the total variability with the first two factors. Elements such as As, Be, Cd, Co, Cr, Se, Tl, U, V had to be eliminated from the data set because of their too low levels (or below LODs). Therefore, the data matrix was constituted by thirteen loadings: Ca, Cu, Fe, K, Mg, Mn, Na, Ni, Pb, Zn, moisture, sugar content and pH. Results are reported in Figure 2.

From this study we can draw some findings. First, the cluster A (honeys from Córdoba and Tucumán provinces) has higher concentrations of Zn and Fe and lower concentrations of Ca, Na and Ni compared to the other groups. Second, the cluster B (Entre Ríos and Santiago honeys) is characterized by higher concentrations of K, Mg, Mn, Cu and Ni and higher pH levels than the other clusters. Moreover, the honeys of the cluster B have lower levels of Zn than the others. Third, the cluster C (Buenos Aires province honeys) showed higher levels of Pb than the other groups and lower levels of sugar, pH and K, Mg, Mn and Fe.

However, these results can be considered as indicative because the DA did not result significant (Montecarlo test RV = 0.126 p = 0.754). Thus, the elements and physiological parameters determined in honey have weak discriminating power. This is of relevance because it further confirms our previous statements [16] that honey is not a reliable biomonitor of environmental contamination, in particular in sites with low levels of contamination (see also refs. [9,45] for discussion). Furthermore, we also conducted the DA on the PCA factors considering the variables ‘unifloral’ and ‘multifloral’ honeys for the elements and physiological parameters determined (results not shown), but also in this case DA resulted not significant (Montecarlo test RV = 0.073 p = 0.309). This further agrees with the statements above reported.

## Conclusions

In general, we can infer that there are no evidences of pollution for the analysed honeys. The analytical results obtained for the Argentine honeys indicate the products’ high quality. In fact, most of the toxic elements were below LODs. The chemometric analysis combining CA, DA and PCA showed their aptness as useful tools for honey’s classification. This study further confirms that the use of honey as biomonitor of environmental contamination is not reliable for sites with low levels of contamination.

Eventually, further research is needed in order to characterize Argentine honeys by means of pattern recognition methods of zones with high production levels and in order to improve their economic interest.

### Experimental

#### ***Samples***

The study was conducted on 16 samples of the typical honeys coming from different production areas in Argentina. Five unifloral and eleven multifloral samples were collected and analysed in duplicate (see Table 4 for description). All collected samples were taken from the local beekeepers’ association with a guarantee of genuineness. All samples were collected, stored in plastic holders and kept at 4–5°C until analysis.

**Table 4:** Argentine honey samples description

**Samples**	**Product**	**Geographical origin**
M1	Eucalipto honey	Colón, Entre Ríos
M2	Chilca honey	Sierras de Córdoba, Córdoba
M3	Algarrobo honey	Aguirre, Santiago del Estero
M4	Mistol honey	Aguirre, Santiago del Estero
M5	Citric honey	Alberdi, Tucumàn
M6	Multifloral honey (organic)	Cruz del eje, Córdoba
M7	Multifloral honey (pasture)	Castelar, Province of Buenos Aires
M8	Multifloral honey	Gualeguaychú, Entre Ríos
M9	Multifloral honey	Concordia, Entre Ríos
M10	Multifloral honey	Mar del Plata, Province of Buenos Aires
M11	Multifloral honey	Berazategui, Province of Buenos Aires
M12	Multifloral honey (creamed)	La Plata, Province of Buenos Aires
M13	Multifloral honey (organic)	Central area, Province of Buenos Aires
M14	Multifloral honey	Tandil, Province of Buenos Aires
M15	Multifloral honey	Central area, Province of Buenos Aires
M16	Multifloral honey	Sierra de los Padres, Mar del Plata, Province of Buenos Aires

#### ***pH, sugar content and moisture***

The pH was assessed by means of a potentiometer utilizing a pH meter Mettler Delta 345 (Mettler Toledo, Milano, Italy) [46]. Sugar and moisture values were determined utilizing a Bertuzzi refractometer (Bertuzzi, Milano, Italy) owing two direct reading displays, for the measurement of sugar content and moisture percent respectively (Chatway method). Total sugar content was expressed as brix degrees [46].

#### ***Determination of mineral elements***

About 0.8 g of fresh honey was treated with 3.5 ml of 70% (w/w) Nitric Acid Suprapur (Merck, Suprapur, Darmstadt, Germany) and 1.5 ml of 30% (w/w) Hydrogen Peroxide Suprapur (Merck, Darmstadt, Germany) in PTFE vessels. The microwave closed digestion system (Milestone, Start D) was used for the mineralization process. The treatment procedure was programmed in four steps with a power of 1200 W applied for 5, 3, 8 and 15 min each respectively. The temperature was 120°C for the first two steps and 200°C for the second two steps. A sample of reference materials and blank was included in each analytical batch. Subsequently, digestion vessels were cooled to room temperature. The final clear solution was made up to 15 mL with DWI water. Digestion methods in biological and environmental matrices were discussed in our previous studies [47,48].

The samples were quantified for 19 elements: As, Be, Ca, Cd, Co, Cr, Cu, Fe, K, Mg, Mn, Na, Ni, Pb, Se, Tl, U, V and Zn, in digested honeys by inductively coupled plasma mass spectrometry [ICP-MSDRC-e (Dynamic Reaction Cell) mod. Elan, Perkin Elmer]. Traceability of results was obtained from the analysis of the certified reference materials NIST-1515 (Apple leaves - National Institute of Standards and Technology) and the Antartic Krill MURST-ISS-A2 (Italian Research Programme in Antarctica). For the MURST-ISS-A2 the mean recovery percentages (five replicates) were: As: 94.1 ± 2.8%; Cd: 93.6 ± 2.8%; Co: 101.2 ± 1.8%; Cr: 98.0 ± 1.1% (not certified, informative concentration); Cu: 101.4 ± 2.0%; Ni: 97.9 ± 2.4%; Pb: 97.0 ± 0.9%; Se: 96.8 ± 2.9% and Zn: 102.1 ± 2.8%. For the NIST-1515 the mean recovery percentages (five replicates) were: Ca: 101.5 ± 2.9%; Fe 100.9 ± 2.9%; K: 98 ± 1.8%; Mg: 102.2 ± 2.7%; Mn: 98 ± 2.2%; Na 99.5 ± 1.8% and V: 101.2 ± 2.8%. Results were in very good agreement with certified values for the tested elements proving good accuracy of the method employed.

All chemicals used in sample treatment were ultra-pure grade (HNO_3_, H_2_O_2_ 30%, Merck, Suprapur, Darmstadt, Germany). Ultra-pure water (Milli-Q system, Millipore Corporation, U.S.A.) was used for all solutions. All glassware was cleaned prior to use by soaking in 10% v/v HNO_3_ for 24 hours before rinsing with Milli-Q water. The standard metal solutions were prepared from stock standard solutions of ultra-pure grade supplied by Merck (Darmstadt, Germany).

The laboratory precision for the whole analytical process was tested by measuring the Relative Standard Deviation (RSD %) of ten replicates for each sample tested (n = 10). The obtained values for the analysed elements were always below 7% proving the good repeatability of the analytical method. For details about uncertainty, precision, accuracy and methods validation see refs [47-50].

#### ***Statistical methods***

Duplicate honey samples were collected at each site. The normality of distributions of the 19 elements determined was tested by using Shapiro-Wilk test [51,52]. Then the homogeneity for the data of the dual collected samples was tested. The t Student test was applied for testing univariate paired comparisons when normal distribution was obtained and the non parametric Wilcoxon rank sum test was applied for the other elements with not normal distribution [53,54]. Results confirmed the data homogeneity (data not shown) and then we merge the data of duplicated samples.

Several approaches can be employed for data analysis in this kind of studies [55,56]. In this work we first applied the hierarchical cluster analysis [57-60] to the data set (16 samples; and 19 elements plus 3 physiological parameters) in order to classify the distribution of the honey samples according to their production areas. The optimal number of clusters was determined by using the hierarchical cut-clustering rule.

The hierarchical clustering by minimum (energy) E-distance method was performed. Dissimilarities are || x-y ||^a^ where the exponent is in the interval (0,2). This function performs agglomerative hierarchical clustering. Initially, each of the n singletons is a cluster. At each of n-1 steps, the procedure merges the pair of cluster with minimum E-distance. The E-distance between two cluster C_i_, C_j_ of size n_i_ and n_i_ is given by

$$e\left(C_{i},C_{j}\right)=\frac{n_{i}n_{j}}{n_{i}+n_{j}}\left[2M_{\mathrm{ij}}-M_{\mathrm{ii}}-M_{\mathrm{jj}}\right]$$

where

$$M_{\mathrm{ij}}=\frac{1}{n_{i}n_{j}}\sum_{p:1}^{n_{i}}{\sum_{q:1}^{n_{j}}\left|\left|X_{\mathrm{ip}}-X_{\mathrm{jq}}\right|\right|}^{a}$$

|| . || denotes Euclidean norm, X_ip_ denotes the p-th observation in the i-th cluster.

Then, discriminant analysis (DA) on principal component analysis (PCA) factors [61] was conducted with the aim to discriminate the elements and parameters classified by cluster analysis (CA). The 13 factors extracted by PCA (i.e. 100% of information) were included in DA. The analysis’ significance was tested by Monte Carlo test (non parametric version of the Pillai’s test) based on 999 replicates [62].

DA and PCA are considered statistical tools capable to reveal structures in environmental data [55,63,64]. In particular, PCA is a statistic technique belonging to the so-called “unsupervised pattern recognition methods,” useful for carrying out exploratory data analysis when there is no preliminary knowledge about the characteristics (i.e., distribution and structure) of the data to be analyzed [65]. DA is a statistic technique belonging to the so-called “supervised pattern recognition methods,” useful for carrying out specific data analysis when a previous unsupervised pattern recognition method, such as PCA, has suggested a potential discrimination among the data. Mainly for this cause, DA is applied to PCA results [66].

Data analysis was performed using the software R version 2.15.2 (2012-10-26) - “Trick or Treat” and the packages Energy, Ade4.

## Competing interests

The authors declare that they have no competing interests.

## Authors’ contributions

MEC conceived of the study and, together with FB, MGF and II participated in its design and drafted the manuscript. MEC, GM and LF coordinated the sampling protocols and the whole analytical procedures. MEC and MGF participated in the design and performed the statistical analysis. This project was based on the ideas and under the guidance and consultation of MEC, MGF, FB and II. All authors read and approved the final manuscript.
